# Describing the communication of autistic people during experiences of distress: A scoping review

**DOI:** 10.1177/13623613261417933

**Published:** 2026-02-14

**Authors:** Karys Oldenburg, Tasia Gibbons, Christie Welch, Ami Tint, Maya Albin

**Affiliations:** 1 School of Rehabilitation Science, McMaster University, Canada; 2 Department of Occupational Science & Occupational Therapy, University of Toronto, Canada; 3 Department of Psychology, University of Calgary, Canada

**Keywords:** autism, communication and language, experiences of distress, speech-language pathology

## Abstract

Autistic people can experience distress for many reasons. Some of these reasons may include unsupportive environments; experiences of trauma; sensory overload; burnout, inertia, meltdown and shutdown; pain; and mental health diagnoses such as anxiety. Little is known about how distress can impact communication and what others can do to provide support in these moments. This scoping review explores how communication is described during experiences of distress for autistic people, including communication partner strategies to support these interactions. Following a comprehensive database search, 18 articles were included in this scoping review. Firsthand perspectives of autistic people were discussed in 10 studies. Distress was most often characterized by instances of anxiety and/or stress. Eleven studies discussed non-spoken methods of communication, including gestures, facial expressions and behavioural manifestations (e.g. hitting, screaming) during experiences of distress. Identified facilitators to communication during experiences of distress included the presence of a calm, supportive environment and communicative aids. Understanding the impact distress has on communication is beneficial to both autistic people and those who support them. This review contributes to the body of literature aiming to better understand and support autistic people during moments of distress.

**Lay abstract:**

Past research talks about (1) how autistic people may communicate and (2) how and why autistic people may experience periods of distress. There is not much research about the way autistic people communicate during periods of distress. We therefore looked at research exploring how autistic people of all ages communicate during periods of distress. Communication includes various methods of sending and receiving information, including spoken and non-spoken communication methods (e.g. observable behaviours, typing, gestures). We explored how often researchers collected firsthand perspectives from autistic people. We also collected information on what made communication easier, or more difficult, during periods of distress. We used a methodological approach called a scoping review to identify and evaluate 18 articles that met our criteria. The most common communication method described in the articles was non-verbal communication, including gestures, facial expressions and observable behaviours such as screaming and hitting. Firsthand autistic perspectives were included in just over half of the studies. Facilitators to communication included a calm, supportive environment and communicative aids relevant to the situation, such as a pain scale. This study will help those who support autistic people, and autistic people themselves, by showing the many ways autistic people may communicate when experiencing distress and describing strategies that can be used to support autistic people in those moments.

Communication serves a variety of purposes, including building relationships, requesting information and conveying information, but also expressing one’s beliefs, feelings, wants, and needs (American Speech-Language-Hearing Association, n.d.-a). While communication can occur using speech, communication also involves augmentative and alternative communication (AAC) methods which can include unaided (e.g. gestures, facial expressions, writing) and/or aided methods (e.g. communication board, speech-generating device) (American Speech-Language-Hearing Association, n.d.-b). The communication needs, abilities and preferences of autistic people are highly variable (Zisk & Dalton, 2019). Some autistic people may communicate primarily through speech, some may use AAC in addition to speech and others may use AAC as their primary method of expression (Donaldson et al., 2021; Zisk & Dalton, 2019). It is estimated that 97 million people worldwide may benefit from AAC use (Beukelman & Light, 2020), with autistic people making up 19% of this population (Creer et al., 2016).

A person’s communicative abilities, needs and preferences may change depending on context and contextual valence (Donaldson et al., 2021; Zisk & Dalton, 2019). For instance, there is a bidirectional relationship between communication and experiences of distress (Allen & Bourhis, 1996; McCabe, 2005). That is, communication difficulties can impact how distress is experienced and expressed, and distress can impact one’s ability to communicate (Donaldson et al., 2021; Zisk & Dalton, 2019). In the latter direction, there is evidence that people with more significant challenges with communication often express themselves using behaviours described as more challenging (Hollo et al., 2014; Chan et al., 2023; Curtis et al., 2018; Matson et al., 2013). Indeed, within the broader intellectual and developmental disabilities (IDD) literature, behaviours that challenge (BTC) are recognized as methods of communication employed when other communication methods are not accessible (Green et al., 2018). As defined by Green and colleagues (2018), BTC are ‘any actions by individuals that are harmful or dangerous to themselves, others, or the environment, or that limit opportunities for inclusion, participation, and integration in their local communities’ (p. S25), including examples such as self-injury, destruction of items or property, actions considered ‘disruptive’ to others such as yelling, or actions which may cause harm to others such as hitting or kicking. Based on research involving autistic participants using minimal to no speech, BTC are similarly found to function as a form of expressive communication during experiences of distress (Chiang, 2008).

Autistic people experience and respond to distress in variable ways, impacted by cognitive, physical and emotional factors; however, current literature and clinical practice is highly reductionistic in nature (Phung et al., 2021). Rather than encompassing a multifaceted or holistic approach, research to date tends to focus solely on individual factors (e.g. behavioural responses, executive function) without consideration of individualized experiences, the role of social and physical environments related to experiences of distress or the interplay between these factors (Hess et al., 2008). The misunderstanding or simplification of autistic people’s experiences of distress may result from a lack of autistic perspectives within the academic literature and the dearth of inclusive research methodologies, particularly for people who do not use speech to communicate (Courchesne et al., 2021).

Currently, much of the literature concerning experiences of autistic people has been obtained by proxy reports (i.e. parent report, and/or professionals working with this population) and exclude autistic people’s firsthand perspectives (Milton & Bracher, 2013). The limited research to date that includes autistic perspectives details how experiences of distress encompass several components, including those that are physical (e.g. sensory regulation, motor control), emotional and cognitive (Belek, 2019; Muris & Ollendick, 2021; Phung et al., 2021). Specific distressing experiences, as described by autistic people themselves, include burnout, inertia, meltdown and shutdown (BIMS) (Phung et al., 2021; Welch et al., 2021). *Burnout* refers to complete exhaustion, characterized by reduced tolerance to stimuli, problems with thinking, and difficulties executing activities of daily living, sometimes leading to withdrawal, distinct from occupational stress or clinical depression (Higgins et al., 2021; Phung et al., 2021; Raymaker et al., 2020). *Inertia* is an inability to initiate movement, including one’s ability to start, stop or transition to a new activity (Buckle et al., 2021; Phung et al., 2021; Rapaport et al., 2024). *Meltdown* refers to a complete state of overwhelm and experience of loss of control (Doherty, 2025; Phung et al., 2021; Soden et al., 2025), while *shutdown* is described as a withdrawal, or feeling frozen, with many struggling to speak or move (Belek, 2019; Paris et al., 2025; Phung et al., 2021; Welch et al., 2021).

Early research on BIMS has found that some autistic people may experience an inability to speak or intermittent speech related to inertia and shutdown (Buckle et al., 2021; Zisk & Dalton, 2019). In addition, communication needs and preferences may also fluctuate for autistic people during experiences of distress (McNaughton et al., 2019). Some autistic people who use both speech and AAC to communicate have been shown to prefer symbol-based AAC during meltdown or shutdown, while others endorse online writing as a preferred method of communication (Donaldson et al., 2021). Corbin (2025), an autistic, semi-speaking AAC user, references ‘expensive speech’ for some autistic people that ‘may be effective but has a significant cost in terms of energy, cognitive resources, or other internal resources’ (Corbin, 2025, p. 1). It has also been reported that speech may be more or less accessible depending on a variety of individual and contextual variables. For instance, Cummins and colleagues (2020) interviewed autistic adults about their communication skills and needs and found that many autistic adults reported communication changes related to anxiety, who the communication partner was, and overwhelm from the communication environment.

## Understanding how communication is described during experiences of distress

Although it is known that autistic people experience distress at higher rates compared to non-autistic people, it is important to understand how communication is described during these experiences (Crane et al., 2019; Lai et al., 2019). Increasing our understanding of how communication is impacted, and described, during experiences of distress is essential to improve our understanding of the communication needs and methods of autistic people, and ensure appropriate support is provided. Exploring this topic in the academic literature is important to illustrate what evidence is already published, what gaps exist in the literature and how existing literature describes communication.

Language shapes how researchers and clinicians provide services and view clients (Lamoureux et al., 2024). For example, language used to describe autistic people and autistic traits has evolved over time and shapes how autistic people are viewed in research and society. There are increasing calls in the literature to move away from binary and reductionistic descriptions of autistic people’s communication, such as labels of verbal/non-verbal (Bottema-Beutel et al., 2025), or high versus low functioning (Alvares et al., 2020). The same discussion of language can be applied to explore how communication, including spoken and non-spoken aspects, is currently described during experiences of distress in the academic literature.

## Objective and research questions

The primary objective of this scoping review was to synthesize available academic literature pertaining to autistic people’s communication during experiences of distress. The focal research question was ‘how is the communication of autistic people described during experiences of distress?’. We also asked two secondary research questions that align with our overarching question, including (a) who is providing the descriptions of communication during distress (e.g. parents, service providers), and to what extent are the perspectives of autistic people represented? and (b) what facilitators and communication supports are described as being effective during experiences of distress? We also sought to capture whether there are discrepancies or similarities in the information provided based on who is describing communication during experiences of distress (i.e. self-report from autistic people, reports by clinicians, families).

## Method

A scoping review was completed in accordance with the Joanna Briggs Institute (JBI) protocol for scoping reviews and under the Preferred Reporting Items for Systematic Reviews and Meta-Analyses Extension for Scoping Reviews (PRISMA-ScR) guidelines (Peters et al., 2020; Tricco et al., 2018). The JBI protocol outlines recommendations to ensure that scoping reviews are rigorous and unbiased (Peters et al., 2020), and PRISMA-ScR guidelines promote consistency of reporting (Tricco et al., 2018). A scoping review was selected to capture the extent of the available literature, synthesize current knowledge and identify any gaps in current understandings and perspectives (Arksey & O’Malley, 2005; Levac et al., 2010; Munn et al., 2018; Peters et al., 2020). Scoping reviews are systematic in nature and allow for a broad exploration of a particular research topic (Armstrong et al., 2011; Tricco et al., 2018). The Population, Concept, Context (PCC) framework was used to define the inclusion and exclusion criteria, outlined below (Peters et al., 2020; Tricco et al., 2018).

### Development of research question and objectives

A multidisciplinary team, consisting of two speech-language pathology graduate students (K.O. and T.G.), a speech-language pathologist (M.A.), an occupational therapist (C.W.) and a psychologist (A.T.), developed the primary and secondary research questions outlined above. We had group discussions about clinical and research gaps in the literature across disciplines. An initial search of OVID MEDLINE was conducted, which concluded that communication during experiences of distress in autistic people had not yet been explicitly explored. All members of our research team are Canadian, cisgender, neurotypical healthcare providers who have clinical and research experiences (i.e. speech-language pathology, occupational therapy, psychology) supporting autistic people across the lifespan. As clinicians and researchers, we take a neurodiversity-affirming, strength-based approach that centres autistic people’s lived experiences using research methods such as co-design and clinical approaches such as family-centred, strength-based services. Our research team embedded opportunities for critical reflexivity about our positionality into our generation of research questions, methods selected, analysis, and interpretation of results, and centred academic and non-academic evidence from autistic people to inform our research question and approach.

### Search strategy to identify relevant studies

#### Database search

The search strategy aimed to identify peer-reviewed, published articles, including both qualitative and quantitative studies with observational, interpretive, experimental and/or quasi-experimental methodologies. An initial search of Ovid MEDLINE was undertaken in April 2024 to identify preliminary search terms. The search terms were refined following consultation with a university librarian (see Supplementary Material 1). Search terms were categorized according to the PCC framework: the population of autistic people (e.g. autism, ASD), concept of communication (e.g. speech, non-verbal, verbal, AAC) and context of experiences of distress (e.g. burnout, meltdown, inertia, shutdown). Databases searched included Ovid MEDLINE, Ovid Embase, APA PsycInfo, CINAHL and Web of Science. Grey literature was not explored, given the intent of this research was to explore and synthesize published, peer-reviewed literature on this topic and to identify any current gaps within the literature. After conducting the search, all citations were uploaded to and managed using Zotero (version 6.0.37). Duplicates were removed using Covidence (Covidence, n.d.).

#### Eligibility criteria

Inclusion and exclusion criteria, outlined below, were determined prior to screening titles and abstracts. Prior to screening full text articles, authors (K.O. and T.G.) refined the inclusion criteria, specifying articles must clearly label an experience as being distressing, using language identified in Table 1. We iteratively discussed the inclusion criteria and established consistency in what would classify an experience as distressing. Due to the subjective nature of distress, a variety of terminology is used within the academic literature (e.g. stress, burnout, inertia, meltdown, shutdown, frustration, anxiety). We included all these terms to capture a broad picture of the concept of ‘distress’ and allow for conclusions to be drawn about the concept rather than one specific term. Distress was defined in our search as discrete instances of distress, not prolonged periods of distress consistent with diagnoses such as depression and/or anxiety. No articles were excluded based on methodology or study design. See Supplemental Material uploaded to Figshare for a detailed list of inclusion/exclusion criteria with examples (Figshare, 2025).

**Table 1.** Inclusion and exclusion criteria.

	Inclusion criteria	Exclusion criteria
Population	Autistic people, including adults and children. Autistic people with any co-occurring diagnoses.	No diagnosis of autism.
Concept	Communication in any capacity, including speaking, minimally speaking, non-speaking and people who use augmentative and alternative communication devices.	No mention of communication.
Context	Specific moments of distress, using the language: *distress/distressing, burnout, inertia, meltdown, shutdown, pain, discomfort, anxiety, suicidal thoughts, depression, anger/frustration.*	Distress was not specific to one instance. Instances in which the author(s) had to assume a situation would be distressing (e.g. being a teen during the COVID-19 pandemic if specific language noted in the ‘inclusion criteria’ was not used).
Other	Looking specifically at the impact of distress on communication.	Grey literature Articles not available in English.

### Data extraction

A data-charting form was created in collaboration with the authorship team. The form included questions that were developed and agreed upon by the team to extract meaningful data and themes from the included studies. The data was organized under corresponding headings in Microsoft Excel, indicating what data belonged in each column. See supplementary materials on Figshare for the complete data extraction form (Figshare, 2025). Direct quotes were extracted with corresponding page numbers when possible. Data extraction was split between authors (K.O. and T.G.) to conduct the initial extraction for each paper. Once the first reviewer had completed their extraction, the other author acted as a second reviewer to double-check the data extracted to ensure that it was correct, and that nothing important was omitted or added in error. The second reviewer made comments in a different colour to track any added or changed information. This process was followed to extract all data. Both authors agreed on over 95% of the data extracted. Any disagreements were resolved through discussion. A third reviewer (M.A.) was available to adjudicate, although this was not needed.

### Synthesis of extracted data

Population, study data, descriptions of distress and descriptions of communication were descriptive in nature and were summarized in table format. The synthesized tables were read and re-read by the authors to identify relevant thematic groupings among concepts in the data using inductive content analysis (Elo & Kyngäs, 2008; Pollock et al., 2023). Open coding was used to identify thematic groupings to answer our research question about the relationship between descriptions of communication and experiences of distress. High-level categories were reviewed with the full authorship team (Elo & Kyngäs, 2008; Pollock et al., 2023). Following discussion, themes were finalized, and descriptive data was synthesized including the study design, population characteristics (e.g. age, gender, co-occurring diagnoses), identified facilitators (e.g. anything that made it easier to communicate during a distressing event), as well as who provided the description(s). This data was extracted and discussed by both reviewers (K.O. and T.G.). Once extraction and data synthesis were completed, the full authorship team discussed the results and collaboratively decided how the results should be reported and connected to clinical implications. Discussions were held during team meetings until consensus among all authors was reached.

## Results

### Database search

**Figure 1.**
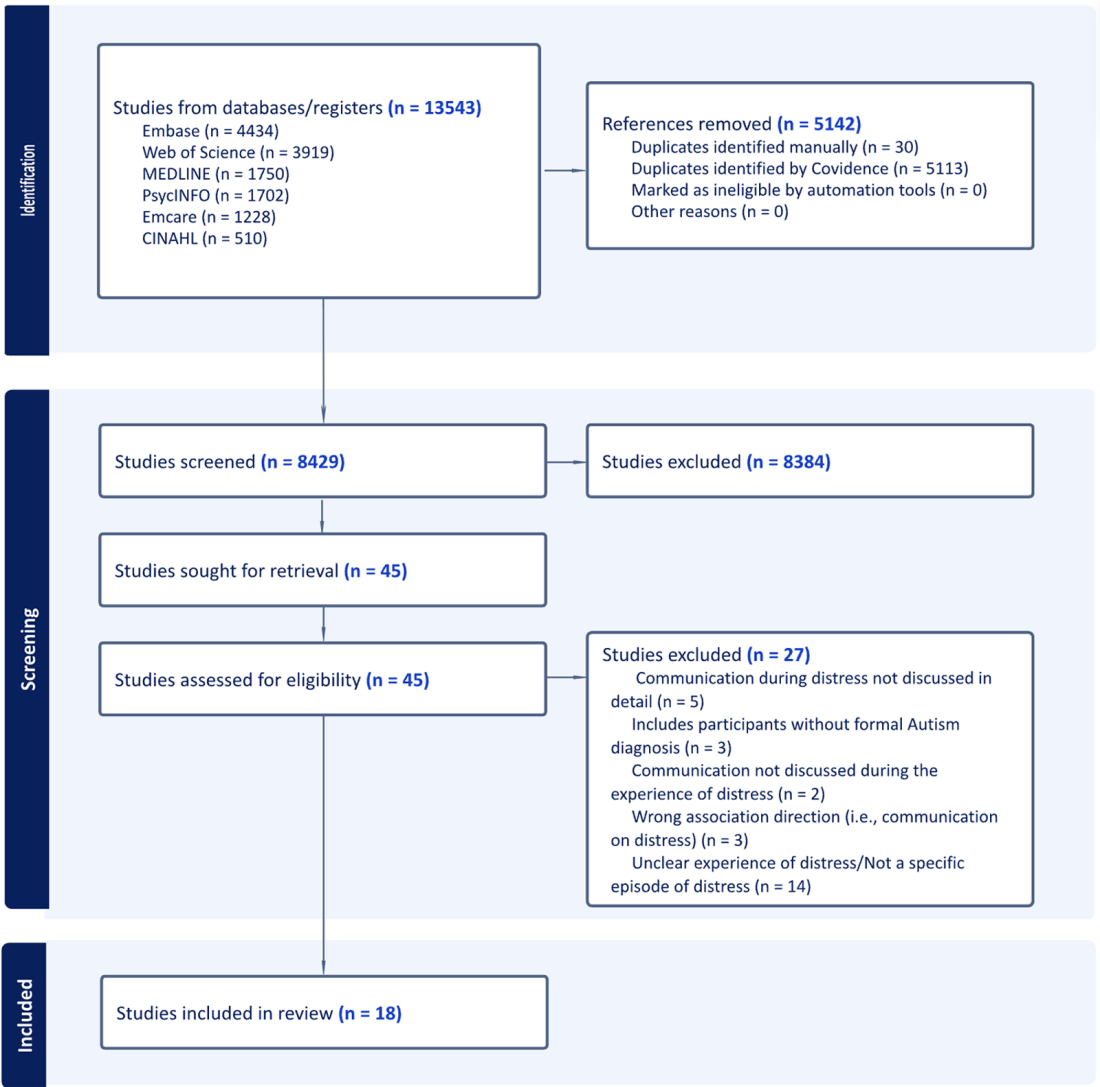
PRISMA-ScR flow chart.

After duplicates were removed, 8429 titles and abstracts were screened. After screening 25 articles together to establish mutual understanding of the inclusion and exclusion criteria, 2 reviewers (K.O. and T.G.) both independently screened 63 titles and abstracts to establish inter-rater reliability, calculated as a percent of agreement between 2 reviewers. Given the high inter-rater agreement (95%), two reviewers proceeded with screening the remaining titles and abstracts independently, divided up evenly. A third reviewer (M.A.) was available to adjudicate disagreements, though this was not needed. In total, 8384 articles were excluded, with many articles excluded for reasons such as focusing on animal/mouse models and genetics or discussing ‘communication’ and ‘distress’ descriptively without exploring the relationship between them. Forty-five full texts were assessed for eligibility, with 27 articles excluded at full text for the reasons detailed in Figure 1, and 18 studies included. One systematic review met the inclusion criteria (Greenwood et al., 2024) but was excluded as all studies in their review were either duplicates of studies we had already included or did not meet our inclusion criteria.

### Study and participant characteristics

Study and participant characteristics are displayed in Table 2. Of the 18 studies, 5 were conducted in the United States, 5 in the United Kingdom, 3 in Australia, 2 in France, 1 in Germany, 1 in Austria and 1 in Canada. In two studies, the country was not specified. One study was conducted across multiple countries (Johnson et al., 2023). Sample sizes ranged from *n* = 1 (e.g. case reports) to *n* = 372 in a survey-based study. Twelve studies included children and/or adolescents, five included adults and one had participants from across the lifespan. Three studies did not specify participants’ sex and/or gender identity, eight included males and females, two included only females, four included only males and one study included participants across the gender spectrum.

**Table 2.** Descriptive population and study data for included studies.

Citation	Country	Study design	Sample size	Age range or descriptors	Sex and/or gender-based information^a^	Psychiatric, developmental and/or physical diagnoses in addition to autism^b^
Ambrose et al. (2024)	Australia	Mixed-methods	45 participants	7–17 years	Male, female, non-binary	Anxiety, ADHD, ID, learning disability, Speech/language delay or disorder, unspecified
Bone et al. (2017)	United States	Quantitative	17 participants^c^	Adolescents	Males and females	Not reported
Bronsard et al. (2010)	France	Quantitative	74 participants^c^	Children and adolescents	Males and females	Not reported
Donovan (2020)	United States, United Kingdom, Australia	Mixed-methods	24 participants	29–65 years	All women	Not reported
Fitzpatrick et al. (2022)	United Kingdom	Quantitative	3 participants	Children	All male	ID
Fogler et al. (2019)	Austria	Qualitative	1 participant	26 years	Man	ADHD, social anxiety, depression, developmental coordination disorder
Heller (2023)	Germany	Qualitative	1 participant	13 years	Boy	Not reported
Holingue et al. (2022)	United States	Qualitative	2 participants^c^	5–25 years	Males and females	Not reported
Johnson et al. (2014)	United States	Qualitative	10 participants	5–12 years	NR	Not reported
Johnson et al. (2023)	Global	Qualitative	10 studies, participants ranging from 2 to 1321 per study	1–21 years	NR	ID, co-occurring gastrointestinal symptoms
Kerns et al. (2023)	Not specified	Mixed-methods	66 participants	6–13 years (children), 14–22 years (youth)	NR	Post-traumatic stress disorder, ID
Lum et al. (2014)	Australia	Quantitative	32 participants	Adults (⩾18 years)	All women	Not reported
Moseley et al. (2019)	United Kingdom	Mixed-methods	103 participants	Adults	Males and females	ADHD, dyslexia, dyspraxia, anxiety, depression
Nader et al. (2004)	Canada	Mixed-methods	21 participants^c^	3–7 years	Boys and girls	Not reported
Ozsivadjian et al. (2012)	United Kingdom	Qualitative	19 participants^c^	7–18 years	Boys and girls	Not reported
Sturrock et al. (2022)	United Kingdom	Qualitative	12 participants	9–14 years	Males and females/boys and girls	Anxiety, ADHD, sensory processing disorder, dyspraxia, dyslexia
Wallace et al. (2021)	United States	Mixed-methods	372 participants	NR^d^	Males and females	Not reported
Weiner et al. (2019)	France	Mixed-methods	1 participant	21 years	Male/man	Not reported

*Notes*. NR = not reported; ADHD = attention-deficit hyperactivity disorder; ID = intellectual disability.

a Terminology listed reflects the terminology used by authors to report demographic information on sex and/or gender in each study. The authors varied in their use of sex and gender terms, and some used the same term to refer to sex and gender. However, terms such as gender/sex should not be conflated and represent distinct concepts.

b Psychiatric, development or other physical health diagnoses were reported for some, but not all, autistic participants.

c Indicates studies including non-autistic participants as well (i.e., as comparison groups). For these studies with non-autistic participants, we have only reported the number of autistic participants.

d All participants in this study were non-autistic parents/health care providers of autistic people. Given that demographic information is only reported for autistic participants, it is listed as ‘NR’ for this study.

### Inclusion of first-person perspectives

Firsthand perspectives from autistic people were included in nine studies, four of which also included proxy reports from parents, caregivers and/or healthcare providers in addition to autistic people’s perspectives. Nine studies included only proxy reports, with three including reports from parents and caregivers, and six describing communication from the perspective of researchers – none of which self-reported being autistic. Studies reporting autistic people’s perspectives tended to report communication difficulties and/or difficulties with verbal expression during distress, as well as what autistic people felt and thought during such instances. For example, autistic people felt misperceived as rude during communicative attempts (Donovan, 2020). Autistic people also noted that they communicated through self-harm, hoping others would notice something was wrong (Moseley et al., 2019). Self-reports from autistic people also described frustrations towards oneself as they struggled to convey their emotions, contributing to a bidirectional relationship between communication and distress (Sturrock et al., 2022). Most studies based solely on proxy reports did not mention difficulties in communication.

### Overview of relationship between distress and communication

**Figure 2.**
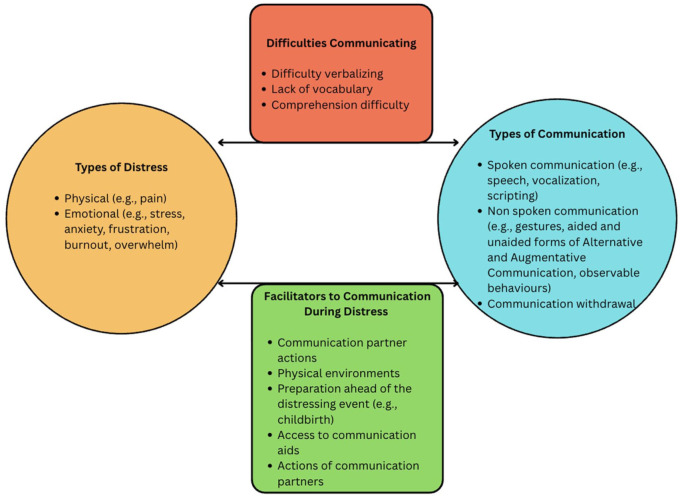
Visual representation of the relationship between communication, experiences of distress and facilitators. This figure visually represents the results of this study, demonstrating the relationship between types of distress experiences, types of communication, and the associated facilitators to communication and difficulties that can occur. Each category contains examples for each domain. The bidirectional arrows demonstrate the relationship between communication and distress, and the facilitators that can support communication during experiences of distress.

Table 3 provides a detailed overview of experiences of distress and how communication is described within the studies. Figure 2 provides a visual representation of the information shared in Table 3 and the relationship between distress and communication.

**Table 3.** Descriptions of distress and communication within included studies.

Citation	People providing description	Distress description	Context of distress	Communication description	Facilitators
Ambrose et al. (2024)	Self-report	Experiences of anxiety	School	Communicative withdrawal; comprehension difficulties; no difficulties	NR
Bone et al. (2017)	Authors	Stress/anxiety	Induced through tasks and measured physiologically (e.g. heart rate)	Higher prosodic variability; vocal changes; rigid/tense; increased rate of speech	NR
Bronsard et al. (2010)	Authors	Stress	Blood draw within a medical centre	BTC (injurious behaviours – i.e. slapping, pinching)	NR
Donovan (2020)	Self-report	Stress, pain, loss of control	Experience of childbirth	Difficulties communicating how they were feeling (i.e. qualifying pain); scripting; communication withdrawal/shutdown; blunt and literal; expression/tone of voice did not correspond with how they were feeling; comprehension difficulties	Being prepared for the stressful situation ahead of time (e.g. having a birth plan)
Fitzpatrick et al. (2022)	Authors	Pain (e.g. dental, gastrointestinal)	4-h period during school day	Non-verbal communication including behaviours (aggression, irritability, self-injury)	Direct intervention targeting expression and localization of pain
Fogler et al. (2019)	Authors	Episodes of anxiety	Returning to group home after visit at parent’s home or after his vocational rehabilitation programme	BTC (tantrum, vocalizations, throwing objects)	NR
Heller (2023)	Authors	Frustration; distress	School/classroom	Verbal (protest); non-verbal (looking towards support staff); withdrawal; vocal changes (higher pitch)	NR
Holingue et al. (2022)	Proxy (parents) + 2 self-report	Pain/discomfort	Gastrointestinal symptoms (nausea, burning, etc.)	Non-verbal (pointing, crossing legs, displays of aggression and / or violence); difficulty verbally communicating presence of symptoms (e.g. descriptive terminology) even for those with fluent speech at baseline	NR
Johnson et al. (2014)	Proxy (parents and providers)	Frustration; burnout	For example, communication difficulty, comprehension difficulty	By parents only: BTC (repetitive behaviours, aggressive behaviours); loud vocalizations; screaming	Visuals; verbal choices; informing the child of what is going to happen; reinforcement; distractions (e.g. toys); counting in a calm voice
Johnson et al. (2023)	5 proxy report (parents, service providers); 5 self-report + proxy report + observer	Pain; pain-related anxiety	Hospital, dentist, home, school, daycare	Verbal responses (speech and vocalizations); Non-verbal responses (facial expressions, gestures, pointing, aggressive behaviours, seeking comfort and closeness, physiological responses, sensory processing)	External aids (iPad); reduce bombardment of questions; descriptions of pain rather than numerical rating
Kerns et al. (2023)	Proxy (clinical providers and researchers)	Stress/anxiety as a traumatic reaction (anxiety and emotional distress)	NR	Perseverative talk; reduced use of communicative language relative to baseline; social withdrawal	NR
Lum et al. (2014)	Self-report	Anxiety; pain; emotional distress	Health care appointment waiting rooms; childbirth	Reduction in capacity to communicate verbally; difficulty communicating pain and needs	Visual pain scales; environmental modification (e.g. reduced volume)
Moseley et al. (2019)	Self-report	Emotional pain; stress; frustration; anxiety	NR	Expression through self-harm	Compassion; patience
Nader et al. (2004)	Proxy (parents) + observation	Pain; distress	Venepuncture	Non-verbal (facial expression)	NR
Ozsivadjian et al. (2012)	Self-report and proxy (parents)	Experiences of anxiety, worry and/or stress	Change or routines; social or language-related triggers; specific fears or phobias; sensory; triggers related to obsessions; triggers related to expectations in performance or organization	BTC (i.e. meltdown, swearing, withdrawal/avoidance; sensory behaviours – i.e. nail biting, obsessive and repetitive behaviours); difficulties with verbal expression	NR
Sturrock et al. (2022)	Self-report	Negative emotions (e.g. anger, stress, annoyance)	Communication difficulties	Difficulties expressing and explaining emotions; lack of vocabulary; shutdown; meltdown; avoidance	Environmental modifications (e.g. headphones, relocating to a calm environment); music to help generate emotional words
Wallace et al. (2021)	Proxy (parents, guardians, caregivers, professionals)	Fear; stress/panic	Police encounters	BTC (pacing, fidgeting, erratic arm movements, loud vocalizations); difficulties communicating (reciprocal communication, answering questions); stuttering; withdrawal	Police training (e.g. increase knowledge of autism, how to promote a safe environment); disclosing the diagnosis; communication tools
Weiner et al. (2019)	Author (observation)	Suicidal ideation	NR	Monotone, rigid communication	NR

*Notes*. NR = not reported; BTC = behaviours that challenge.

### Descriptions of distress

In 11 studies, distress was characterized by instances of anxiety and/or stress. Pain was also a common description of distress, discussed in six studies. Experiences of physical pain included gastrointestinal pain (e.g. Holingue et al., 2022) and pain during intravenous administration (e.g. Nader et al., 2004). Other studies looked at general physical pain while discussing how autistic people alert others to their pain. In over half of the studies, more than one description of distress was provided. For example, Wallace and colleagues (2021) used the terms fear, stress and panic, while Moseley and colleagues (2019) used the terms emotional pain, stress and frustration. Common contexts in which experiences of distress were described include hospitals, healthcare appointments and schools.

### Context of distressing experiences

See Table 3 for details on descriptions of distress and communication for each study. Of the six studies reporting autistic adults’ experiences of distress, the context of the distressing event, when specified, included interactions with health care professionals or police, or events known to be distressing to the specific person (e.g. returning to group home). In the studies including children and adolescents, descriptions of distress and the context in which they occurred varied. Distress occurred in school context in three studies, multiple contexts (e.g. home, daycare) in one study, venepuncture (e.g. blood draws) in two studies and unspecified in five studies. In one study, the distress occurred in a specific anxiety-inducing task (e.g. presentation).

### Description of communication during experiences of distress

The terminology used to describe communication was variable, including verbal expression, non-verbal expression, BTC, communicative withdrawal, reduced communicative capacity and suprasegmental changes. Figure 2 provides a visual representation of the relationship between types of distress experiences, types of communication and the associated facilitators to communication and difficulties that can occur.

#### Non-verbal communication

Eleven studies described non-verbal communication (i.e. expressive communication not involving the use of any verbal output, including facial expressions, gestures and BTC, during experiences of distress). BTC, behaviours that result in potential harm to oneself or others, were identified as forms of expression in nine studies. Behaviours were described as ‘aggressive’ (Fitzpatrick et al., 2022; Holingue et al., 2022; Johnson et al., 2014, 2023), ‘challenging’ (Ozsivadjian et al., 2012), ‘injurious/self-harm’ (Bronsard et al., 2010; Fitzpatrick et al., 2022; Moseley et al., 2019) including head banging or self-hitting (Kerns et al., 2023), or ‘tantrum/meltdown’ (Fogler et al., 2019; Sturrock et al., 2022). Of the nine studies that described communication through BTC, six looked at children and adolescents, people of all ages or did not specify the age range. Seven studies discussed a withdrawal from and/or avoidance of, either the use of communication and/or the social context/interaction, due to the distress. Among the three studies that looked exclusively at the school context, two noted communicative withdrawal among other descriptions of communication (Ambrose et al., 2024; Heller, 2023). Other communicative behaviours were seeking comfort, and repetitive behaviours, including examples such as hand flapping and rocking.

#### Verbal communication

Verbal communication methods were described in six studies, with methods including speech (i.e. spontaneous production of original words and phrases), loud vocalizations (i.e. the production of sounds that do not represent distinctive words), stuttering and using scripted/echolalic phrases. Three studies described speech using the terminology ‘blunt and literal’ (Donovan, 2020), and ‘rigid’ (Bone et al., 2017; Weiner et al., 2019). Seven studies discussed a reduction in verbal communicative capacity during experiences of distress. Out of the seven studies that took place in a healthcare setting, four explicitly mentioned that communicative capacity was reduced and/or that participants had difficulties localizing, verbalizing and/or quantifying pain or the presence of a symptom. Comprehension difficulties during experiences of distress were reported in two studies. Three studies reported suprasegmental (i.e. features of speech beyond individual sounds) changes to verbal communication, including a mismatch between tone of voice and emotion, prosodic variability, increased speech rate and vocal changes such as higher pitch.

#### Relationship between types of distress and descriptions of communication

We analysed how communication was described during instances of physical distress (e.g. pain) compared to emotional distress. In the six studies investigating physical distress related to pain, most reported non-verbal forms of communication such as the use of facial expressions and gestures, difficulty communicating and/or verbalizing pain, and BTC. Verbal communication and communication withdrawal were the least reported. Sixteen studies described communication during instances of emotional distress such as anxiety, burnout and/or frustration. In contrast to studies involving physical distress, studies discussing emotional/psychological distress described communication withdrawal and verbal communication most often (i.e. seven studies). BTC were also described in seven of the studies. Non-verbal communication methods were mentioned less often (i.e. three studies). While these are interesting relationships to consider, findings should be interpreted with caution as studies describing emotional distress were more than two times as frequent as studies mentioning physical distress. Many studies also reported on both physical and emotional experiences of distress, as outlined in Table 3.

### Facilitators to communication during experiences of distress

No studies explicitly discussed barriers to communication during distress, and seven studies discussed facilitators. Within a healthcare setting, recommended facilitators included being prepared ahead of time by having a plan in place (Donovan, 2020), using external aids such as an speech-generating device or visual pain scale, and cultivating a supportive environment by limiting bombarding questions and relocating to a calm and quiet space (Johnson et al., 2023; Lum et al., 2014). When distress was expressed through self-injurious behaviours, one study identified the importance of displaying patience and compassion (Moseley et al., 2019). Another study that focused on children who primarily communicate distress through self-injurious and aggressive behaviours discussed direct interventions to help describe or localize pain to provide specific support (Fitzpatrick et al., 2022). Communication partner training was also identified as a facilitator. In their study investigating the experience of autistic people during police encounters, Wallace et al. (2021) mentioned that professional training opportunities could be beneficial, helping police officers better understand how to promote a more comfortable interaction during a stress-inducing event.

## Discussion

Autistic people communicate through various methods, and experiences of distress may result in, or exacerbate, communication difficulties (Donaldson et al., 2021; Zisk & Dalton, 2019). Descriptions of communication, or identification of communication attempts, differ based on the person providing the description (i.e. autistic people, parents or professionals), with first-person descriptions from autistic people often providing more detailed insight into the relationship between communication and experiences of distress. Our results provide an overview of how the relationship between distress and communication for autistic people is described in extant academic literature and highlights potential roles and responsibilities for communication partners of autistic people.

### Barriers and facilitators to communication during experiences of distress

Communication partners (i.e. service providers, family, friends) can support autistic people during experiences of distress themselves and support communication during those experiences. Facilitators identified in our review include visual aids and supports (e.g. pain scales, AAC), reducing communicative demands (e.g. by offering choices) and adjusting the environment to be more calming and supportive (Johnson et al., 2014; Lum et al., 2014). These suggestions are consistent with existing evidence, including communication improvements for autistic youth when wearing headphones in a loud environment (Muris & Ollendick, 2021), or visual support and AAC facilitating communication for both speaking and non-speaking people (Keville et al., 2023; Zisk & Dalton, 2019).

Importantly, communication partners can also negatively impact both experiences of distress and effective communication. For example, a lack of access to communication aids or awareness on the part of the communication partner could act as a communication barrier. Attitudinal barriers also include misconceptions surrounding AAC, such as the belief that it is only beneficial for non-speaking people (Zisk & Dalton, 2019). To facilitate improved support for autistic people during periods of distress, parents and professionals should be aware that AAC can benefit people with differing communication needs, including people who use speech to communicate (Donaldson et al., 2021; McNaughton et al., 2019). Parents and professionals should also be mindful that different communication methods may be more accessible/preferable depending on the context (Donaldson et al., 2021). When discussing AAC and a client’s language abilities, clinicians and researchers should be thoughtful about language, specifically the recommendation to use ‘non-speaking’ instead of ‘non-verbal’ as a descriptor for someone’s communication (Bottema-Beutel et al., 2025).

A lack of empathy and/or misunderstanding from others can also be a barrier to communication during experiences of distress (Doherty, 2025; Phung et al., 2021; Rapaport et al., 2024; Raymaker et al., 2020; Soden et al., 2025). Potential solutions include supporting the training of neurodivergent healthcare providers who may better understand neurodivergent patients (Oates & Bean, 2023), or education for neurotypical healthcare providers on the mutual responsibility of communication and repairing communication breakdowns consistent with the double empathy problem (Milton, 2012; Milton et al., 2022). A potential framework for this training is the intervention model communication partner training (CPT), which is an evidence-based method to support dyadic communication interactions that has emerging applications for autistic people (Albin et al., 2025; Clarke & Fung, 2022; Norris et al., 2024).

### Behaviours that challenge as communication

Many studies described BTC as a form of communication during experiences of distress, though this differed according to the person providing the description. For instance, Johnson and colleagues (2014) report how parents identified ‘aggressive behaviour’ as a form of communication for their autistic child, while health care providers attributed it to the child having higher support needs and did not mention their communicative function. Communicative functions of BTC are variable and may include requesting and protesting (Chiang, 2008), expressions of sensory overload and/or emotions such as anger and seeking comfort (Ferguson, Russell, et al., 2025; Ferguson, Spackman, et al., 2025; Freeman et al., 2002; Ho et al., 2012; Richards et al., 2017; Summers et al., 2017).

Furthermore, given that some autistic people may not be able to offer additional context pertaining to the BTC (i.e. via AAC or spoken communication), the function or intention of the behaviour may be speculative when interpreted by proxy reporters. As such, it is important to be aware of the communication function of BTC and recognize that people require support rather than judgement or reductionistic ‘behaviour management’. This recommendation is consistent with universal trauma and violence informed care approaches, which are valuable as a universal approach regardless of whether the intent or function of the behaviour is clarified (Wiseman-Hakes et al., 2025).

Awareness of the ways in which autistic people communicate, particularly during periods of distress, is important as communication breakdowns, BTC, being misunderstood can lead to delayed support, as well as a mismatch in the support provided compared to what is being experienced, as seen in pain management within the healthcare system (Donovan, 2020; Johnson et al., 2023). Experiences of distress and communication abilities are often inextricably linked, whereby the identified communication facilitators can help autistic people get their needs met and mitigate distress, which, in turn, can support communication. This relationship is well illustrated in SLP literature that discusses how supporting functional communication (e.g. determining the communicative function of the behaviour in question and providing support) can reduce instances self-injurious behaviours, further speaking to the close relationship between these two entities (Carr & Durand, 1985; Gerow et al., 2019).

### Bi-directional relationship between experiences of distress and communication

While all the included studies discussed how communication may be impacted by experiences of distress, some studies also discussed how experiences of distress can be exacerbated or changed due to difficulties with communication. For example, Sturrock and colleagues (2022) found that autistic participants reported increased frustration because of difficulties communicating their emotions, which led to further distress. Johnson and colleagues (2014) described how autistic children’s feelings of frustration and burnout resulting from communication difficulty led to ‘repetitive’ or ‘aggressive’ behaviours as well as loud vocalizations/screaming from the perspective of their parents. These findings reflect a cyclical pattern wherein difficulty communicating or being misunderstood can lead to distress, which can further increase communication difficulties or communication breakdown. It is important for clinicians and researchers to understand this relationship and approach communication compassionately rather than with judgement.

Our results also included how some autistic people may experience and express pain, and how that impacts their communication. Included studies investigating pain and/or physical discomfort in autistic participants noted a difficulty in verbally communicating pain, a reduction in spoken communication, and non-spoken communication being most common. Misinterpretation of these communication attempts may contribute to false stereotypes that autistic people are insensitive or have reduced sensitivity to pain, rather than the evidence that autistic people express pain differently than a neurotypical people (Allely, 2013). This idea persists, though a systematic review called this stereotype into question, finding that autistic people generally do not have different thresholds or responses to pain compared to non-autistic people (Moore, 2015).

Autistic people should be centred in research and clinical resources that describe the relationship between experiences of distress and communication. Half of the studies in our review included autistic perspectives, with these studies often providing a more descriptive and nuanced descriptions of communication as compared to proxy reports, often from neurotypical people. Our findings are consistent with recent research on how autistic people describe experiences of distress and their communication, which found that these experiences are multifaceted and neurotypical understandings are often reductionistic (Phung et al., 2021). This finding underscores the importance of centring autistic people in research on autistic experiences to gain a rich, nuanced understanding of their experiences and avoid perpetuating incorrect or reductionistic assumptions.

### Limitations and future directions

Our study has several limitations. Distress is a highly individualized and nuanced concept based on one’s own lived experiences (Adame & Hornstein, 2006; Buckle et al., 2021; Cromby et al., 2013; Moult et al., 2020; Raymaker et al., 2020; Welch et al., 2021). The absence of a unifying taxonomy creates challenges to identifying and synthesizing all relevant literature on experiences of distress. Given this inherent subjectivity, our selection criteria necessitated experiences to be labelled as ‘distressing’ or as causing a distressing emotion such as fear, anxiety or discomfort. Our search terms were based on our collective clinical experience, searches of the academic literature and consultation with a university librarian, but articles may have been missed if distress was described using different terms than our search included.

We also recognize that terminology referring to distress and communication in non-academic literature may differ. There is emerging research aiming to explore and describe experiences of distress from autistic perspectives (Belek, 2019; Buckle et al., 2021; Higgins et al., 2021; Raymaker et al., 2020; Welch et al., 2021), including specific concepts such as BIMS (Phung et al., 2021). As this research continues to be conducted, the relationships between distress, including distinct types of distress, and communication for autistic people may be further elucidated, and additional ways to refer to, or conceptualize, distress and/or communication may emerge.

Our study explored how communication during distress is described in the published academic literature. However, these results should not be extrapolated to represent perspectives that may be present in the grey literature. Academic literature does not represent the wide variety of experiences of autistic people given that autistic people with intellectual disability, AAC users and multiply marginalized (i.e. racialized, non-English speaking, lower socioeconomic status) autistic people are often left out of research studies (Bertilsdotter Rosqvist et al., 2019; Dee-Price et al., 2020; Fletcher-Watson et al., 2021; Walsh et al., 2024). Participants who are selected, or self-select, to participate in research may also represent a specific portion of the community. Future research could explore non-academic sources such as blogs, social media or other forums describing the relationship between communication and experiences of distress. Grey literature, such as blog posts or podcasts as have been seen in phenomenological studies (Gillespie-Lynch et al., 2014; Welch et al., 2019, 2021, 2022), may also include more firsthand perspectives from autistic people compared to academic literature which contains mostly proxy reports.

Future studies could explore the relationship between communication and experiences of distress using an intersectional lens, considering how more individualized characteristics (e.g. social determinants of health, experiences of privilege and oppression, baseline distress tolerance, emotion regulation) may influence communication during periods of distress (Mallipeddi & VanDaalen, 2022). Previous negative experiences in settings such as schools, hospitals or police encounters may also influence how one communicates during moments of distress (Beck, 2024; Kerns et al., 2015, 2022). An intersectional approach should also consider how factors such as gender, age, ethnicity or co-occurring diagnoses impact how communication is described during experiences of distress, and what supports are provided (Keane & Kocsis, 2025; Mallipeddi & VanDaalen, 2022; Pope et al., 2022). Intersectionality should be considered for any studies collecting data from autistic participants and the results of any literature review within the broader landscape of who is typically included, or left out, of autism research (Lovelace et al., 2021; Malone et al., 2022; Maye et al., 2022; Russell et al., 2019).

## Conclusions and clinical implications

Communication is a shared, bidirectional responsibility for both communication partners (Milton, 2012; Milton et al., 2022) and a basic human right (McLeod, 2018; United Nations, 1948). Communication partners, such as professionals working with autistic people, family members or friends, can benefit from understanding how experiences of distress impact communication, and how communication challenges can lead to or worsen experiences of distress. This is especially true for those working in settings where distress is more commonly experienced, including, but not limited to, health care workers, teachers and law enforcement. Findings from this study suggest that professionals can provide better support by approaching autistic people’s experiences of distress with empathy, curiosity and a willingness to invest time in understanding a person’s unique experiences of distress, variability in communication and how these things may interact. In addition, practical strategies such as visual aids and supports (e.g. pain scales, AAC) and reducing communicative demands may be helpful. It is important to continue to amplify first-person autistic perspectives in this work and support non-autistic communication partners to better understand how support can best be provided during experiences of distress.

## Supplemental Material

sj-docx-1-aut-10.1177_13623613261417933 – Supplemental material for Describing the communication of autistic people during experiences of distress: A scoping review Supplemental material, sj-docx-1-aut-10.1177_13623613261417933 for Describing the communication of autistic people during experiences of distress: A scoping review by Karys Oldenburg, Tasia Gibbons, Christie Welch, Ami Tint and Maya Albin in Autism
