# Supplementary material for: Describing the communication of autistic people during experiences of distress: A scoping review
Source: Autism. 2026 Feb 14;30(4):884–900. doi: 10.1177/13623613261417933 (PMC13005890; doi:10.1177/13623613261417933)
Supplement: sj-docx-1-aut-10.1177_13623613261417933 – Supplemental material for Describing the communication of autistic people during experiences of distress: A scoping review [file sj-docx-1-aut-10.1177_13623613261417933.docx]

**Supplementary Material 1**

**Search terms and search strategy, for OVID MEDLINE.**

| Population | Concept | Context |
| --- | --- | --- |
| autis*.mp.  Exp Autism Spectrum Disorder/  Autistic Disorder/  Asperger’s Syndrome/ | communicat*.mp.  interpersonal Communication/  Verbal communication/  Nonverbal communication/  ((intermittent or insufficient or unreliable) adj2 (speech or communicat* or language)).mp.  (AAC or Alternative Augmentative Communication or Partial AAC use* or gesture*).mp.  (speech or speak*).mp.  Exp Verbal behaviour/  Speech/  Speech intelligibility/  talk*.mp.  mutism.mp.  mute.mp.  mutism/  echolalia/ | distress*.mp.  Psychological distress/  Psychological stress.mp.  stress, psychological/  burnout, psychologic/  emotional exhaustion/  meltdown*.mp.  shutdown*.mp.  inertia.mp.  burnout.mp.  anxi*/  ((challeng* or attention-seek* or aggressi* or self-injurious or violent* or distrupt* or external* or explosiv*) adj2 behav*).mp.  problem behavior/ or self-injurious behavior/  cataton*.mp.  catatonia/  Executive d?sfunction*.mp.  tantrum*.mp.  Autistic regression.mp.  cris?s.mp.  panic*.mp.  Exp fear/  Panic/  elop*.mp. |

*Note.* Search terms were reconfigured slightly to align with specific database requirements*.*
